# Fetal muscle extract improves muscle function and performance in aged mice

**DOI:** 10.3389/fphys.2022.816774

**Published:** 2022-10-07

**Authors:** Hiu Tung Jessica Lo, Tsz Lam Yiu, Yujia Wang, Lu Feng, Gang Li, May Pui-Man Lui, Wayne Yuk-Wai Lee

**Affiliations:** ^1^ Musculoskeletal Research Laboratory, Department of Orthopaedics and Traumatology, Faculty of Medicine, The Chinese University of Hong Kong, Prince of Wales Hospital, Hong Kong, China; ^2^ Li Ka Shing Institute of Health Sciences, The Chinese University of Hong Kong, Hong Kong, China; ^3^ Stem Cells and Regenerative Medicine Laboratory, Lui Che Woo Institute of Innovative Medicine, The Chinese University of Hong Kong, Prince of Wales Hospital, Hong Kong, China; ^4^ SH Ho Scoliosis Research Laboratory, Joint Scoliosis Research Centre of the Chinese University of Hong Kong and Nanjing University, Department of Orthopaedics and Traumatology, The Chinese University of Hong Kong, Hong Kong, China; ^5^ Alis Pharma Limited, Hong Kong, China

**Keywords:** sarcopenia, aging, muscle, muscle extract, iTRAQ

## Abstract

**Background:** Loss of skeletal muscle mass and function is one of the major musculoskeletal health problems in the aging population. Recent studies have demonstrated differential proteomic profiles at different fetal stages, which might be associated with muscle growth and development. We hypothesized that extract derived from fetal muscle tissues at the stage of hypertrophy could ameliorate the loss of muscle mass and strength in aged mice.

**Methods:** To allow sufficient raw materials for investigation, skeletal muscle extract from fetal sheep at week 16 of gestation and maternal tissue were used in the present study. iTRAQ (isobaric tags for relative and absolute quantitation) and KEGG pathway analyses identified differentially expressed proteins in fetal sheep muscle extract vs. adult sheep muscle extract. Effects of FSME and ASME on human myoblast proliferation were studied. To examine the effect of FSME *in vivo,* C57BL/6 male mice at 20 months of age were subjected to intramuscular administration of FSME or vehicle control for 8 weeks. A grip strength test and *ex vivo* muscle force frequency test were conducted. Finally, serum samples were collected for multiplex analysis to determine potential changes in immunological cytokines upon FSME injection.

**Results:** Compared with ASME, 697 and 412 peptides were upregulated and downregulated, respectively, in FSME, as indicated by iTRAQ analysis. These peptides were highly related to muscle development, function, and differentiation from GO enrichment analysis. FSME promoted cell proliferation of myoblast cells (+300%, *p* < 0.01) without causing significant cytotoxicity at the tested concentration range compared with ASME. After 8 weeks of FSME treatment, the percentage of lean mass (+10%, *p* < 0.05), grip strength (+50%, *p* < 0.01), and ability in fatigue resistance were significantly higher than those of the control group. Isometric forces stimulated by different frequencies were higher in the control group. Histologically, the control group showed a larger cross-sectional area (+20%, *p* < 0.01) than the FSME group. The multiplex assay indicated that FSME treatment did not lead to an elevated circulatory level of inflammatory cytokines. Of note, after FSME treatment, we observed a significant drop in the circulating level of IL-12 (p40) from 90.8 ± 48.3 pg/ml to 82.65 ± 4.4 pg/ml, G-CSF from 23476 ± 8341.9 pg/ml to 28.35 ± 24.2 pg/ml, KC from 97.09 ± 21.2 pg/ml to 29.2 ± 7.2 pg/ml, and RANTES from 325.4 ± 17.3 pg/ml to 49.96 ± 32.1 pg/ml.

**Conclusion:** This is the first study demonstrating the beneficial effect of fetal muscle extract on muscle health in aged mice. Further analysis of the active ingredients of the extract will shed light on the development of a novel treatment for sarcopenia.

## 1 Introduction

By 2050, the global population of older people aged 60 or above is expected to be more than double its size in 2015 (Nations, 2013). Age-associated musculoskeletal disorders are inevitable, thus leading to an increasing demand for primary healthcare and long-term care in most countries. Sarcopenia is attracting research and public attention because of its significant impact on healthcare and socioeconomic systems. Sarcopenia is a common geriatric muscular disease characterized by a decrease in muscle mass, strength, and performance, which increases the risk of all-cause mortality (Cruz-Jentoft et al., 2010). Physical training is a well-recognized regime to reduce muscle loss during aging, but the limited efficacy on bedridden people has prompted the search of an equally, if not more, effective pharmacological approach (Fielding et al., 2011). Although combined exercise was found to be an effective tool showing preventive and therapeutic effects on age-related sarcopenia, forms of exercise and the actual cellular and molecular mechanisms responsible for protective effects have not yet been studied thoroughly and no census found until now (Yoo et al., 2018). Supplements, such as vitamin D and omega-3 fatty acids, have been developed as alternative options to treat age-related primary sarcopenia, but their clinical efficacy remains to be ascertained (Lo et al., 2020). With no effective treatment after diagnosis, sarcopenia can deteriorate older adults’ quality of life (Lo et al., 2020). In order to achieve active aging proposed by the World Health Organization, there is a growing scientific and public interest to develop effective approaches to counteract the effects of sarcopenia in order to maintain functional independence, which extend longevity and enhance one’s quality of life (World Health, 2002).

Previous studies have demonstrated that young blood is able to alleviate age-associated pathological conditions (Ludwig and Elashoff, 1972; Conboy et al., 2005; Castellano et al., 2015; Rebo et al., 2016). Through the use of the parabiosis technique, growth differentiation factor 11 (GDF11) and C-C motif chemokine 11 (CCL11) were found to be differentially expressed in young and aged blood. Although the effects of GDF11 and CCL11 remain debatable, these findings, nonetheless, have inspired subsequent research on novel therapeutics for age-associated disorders (Villeda et al., 2011; Sinha et al., 2014; Nations, 2017). These studies indicate that the tissue/blood extracted from young and old might have different molecular and proteomic profiles. Similar to the parabiosis model, pregnancy can also be deemed as a blood factor exchange model that actively takes place in the placenta where the maternal circulation is in close contact with the fetal circulation (Gielchinsky et al., 2010). Using aged conceived mice, Michaeli et al. found an improvement in muscle regeneration post-injury in comparison to unconceived controls, supporting the idea that factors generated by the fetus can have a beneficial effect when applied to aged organisms (Falick Michaeli et al., 2015).

During fetus muscle development, insulin-like factor (IGF) is elevated in order to promote the growth of muscle cells and cell proliferation by activating the MAP kinase-dependent signaling (MAPK) and the PI3K/AKT pathways (Fowden, 2003). With these, we hypothesized that the fetal muscle extract undergoing rapid muscle development consists of a unique proteomic profile (Nieman et al., 2001; Suzuki et al., 2006; Neubauer et al., 2014), making them attractive sources to develop potential therapeutics for muscle aging. In this proof-of-concept study, fetal muscle tissue derived from large animal fetus, such as sheep, was employed in this study to ensure sufficient raw materials for extraction and administration (Xu et al., 2017; Liu et al., 2019). Furthermore, in view of its clear transcriptome profile and well-understood differential expression of molecular markers related to muscle development (Arora et al., 2019), muscular extract derived from fetal sheep (FSME) and adult sheep (ASME) were used as surrogates to demonstrate whether FSME is able to promote myoblast proliferation *in vitro* and improve muscle performance *in vivo*.

## 2 Methods and materials

### 2.1 Animals

**FIGURE 1:**
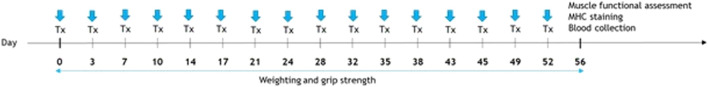
Schematic illustration of an animal study.

C57BL/6 male mice (20 months old, body weight of 35–50 g) were obtained from The Jackson Laboratory. Mice were then randomized into two groups receiving an intramuscular injection in the gastrocnemius of 30 mg/ml of FSME (treatment group, *n* = 5) or phosphate buffer saline (PBS group, *n* = 5), and injections were subsequently performed twice a week for 8 weeks. The timeline is presented in Figure 1. Mice were housed in cages at 21°C and constant humidity with a standard 12:12 h light/dark cycle. Ethics approval was obtained for this animal experiment from the Ethics Committee of the Chinese University of Hong Kong (AEEC Ref No. 18–132-ICP).

### 2.2 Preparation of fetal sheep-derived muscle and adult sheep-derived muscle extracts

A cesarean section was performed on a 16-week pregnant sheep to isolate the fetus. Gastrocnemii of 16-week-old sheep fetus and adult sheep were isolated. An equal volume of sterile PBS (1 ml/1 g) was used as a homogenization buffer. Gastrocnemii from fetal and adult sheep were cut into small pieces before being homogenized with a pestle homogenizer on ice (33 rpm, 1 min) to obtain FSME and ASME homogenate. The homogenates were centrifuged at 5,000 x g for 15 min at 4°C to remove tissue debris, followed by filtration through a 70 μm cell strainer and a 0.22 µm filter to remove cells and debris and to retain bioactive components. The final products, i.e., the FSME and ASME, were stored at -80°C until use. Total protein concentration was measured by using the Pierce BCA assay kit (Thermo Fisher United States . Cat. 23225) to standardize the amount for cellular and animal studies*.*

### 2.3 Protein isolation and iTRAQ labeling

To process the samples for iTRAQ analysis, proteins were extracted from fetal muscle and adult muscle using lysis buffer (8 M Urea, 40 mM Tris-HCl with 1 mM PMSF, 2 mM EDTA, and 10 mM DTT, pH 8.5) in accordance with the BGI established protocol. Protein samples derived from fetal sheep muscle extract (FSME) and adult sheep muscle extract (ASME) were first quantified with Bradford assay, and 30 µg of the samples were resolved using SDS-PAGE and stained with Coomassie blue for quality assessment. The protein samples were subsequently diluted 4 times using urea free lysis buffer to attain a urea concentration of 2 M and subjected to trypsin digestion (Trypsin Gold (Promega, Madison, WI, United States)) overnight at 37°C. Peptide samples were desalted with Strata X C18 column (Phenomenex) and vacuum-dried according to manufacturer’s protocol and then redissolved in 30 µL 0.5 M TEAB provided by using the ITRAQ Reagent 8-plex Kit. Peptide labeling was performed by the iTRAQ Reagent 8-plex Kit (SCIEX, United States).

### 2.4 Peptide fractionation and LC-MS/MS analysis

The labeled sample peptides were combined and subjected to high-performance liquid chromatography (Shimadzu, Kyoto, Japan) for fractionation. The peptides were reconstituted with buffer A (5% ACN, 95% H2O, pH adjusted to 9.8 using ammonia) and loaded onto a 5 µm particle column (Phenomenex) and resolved at a flow rate of 1 ml/min with a gradient of 5% buffer B (5% H_2_O, 95% ACN, pH adjusted to 9.8 with ammonia) for 10 min, 5–35% buffer B for 40 min, 35–95% buffer B for 1 min. Elution of peptide samples was monitored by measuring absorbance at 214 nm, and fractions were collected every 1 min. The eluted peptides are pooled into 20 fractions and vacuum-dried.

The eluted peptide fractions were first resuspended in buffer A (2% ACN and 0.1% FA in water) and centrifuged at 20,000 g for 10 min. The supernatant was loaded onto a C18 trap column at a 5 µL/min rate for 8 min using a LC-20AD nano-HPLC instrument (Shimadzu, Kyoto, Japan) auto-sampler. The resolved samples were subjected to tandem mass spectrometry scan analysis. MS scan data were acquired with the following MS conditions: an ion spray voltage of 2,300 V, curtain gas of 30, nebulizer gas of 15, and interface heater temperature of 150 °C. A high-sensitivity mode was used for the whole data acquisition. The accumulation time for MS1 was 250 ms, and the mass range was from 350 to 1,500 Da. Based on the intensity in the MS1 survey, as many as 30 product ion scans were collected if exceeding a threshold of 120 counts per second (counts/s) and with charge-state 2+ to 5+, dynamic exclusion was set for 1/2 of the peak width (12 s). For iTRAQ data acquisition, the collision energy was adjusted to all precursor ions for collision-induced dissociation, and the Q2 transmission window for 100 Da was 100%.

### 2.5 Database search, protein identification, and quantification

The MS/MS data were searched against the Ovis_aries (27620 sequences) database for peptide identification and quantification using the Mascot 2.3.02. For protein identification, a mass tolerance of 0.1 Da for fragment mass tolerance and 0.05 Da for peptide mass tolerance. Oxidation (M) and iTRAQ8plex (Y) were the potential variable modifications, and Carbamidomethyl (C), iTRAQ8plex (N-term), and iTRAQ8plex (K) were fixed modifications. The charge states of peptides were set to 2 + to 5+. Software Mascot was used for bioinformatics analysis against the selected protein database Uniport. The protein quantification was performed by automated software IQuant (Wen et al., 2014). The peptide number contained at least one unique spectra. The differentially expressed proteins were defined with a >1.2 or <0.83-fold change (mean value of all comparison groups), and *p* value (*t*-test of all comparison groups) less than 0.05 was considered as statistically significant. All the proteins with a false discovery rate (FDR) less than 1% were proceeded with downstream analysis, including Gene Oncology (GO), cluster of Orthologous Groups of proteins, and KEGG pathway analysis.

### 2.6 3-(4,5-Dimethylthiazol-2-yl)-2,5-diphenyltetrazolium bromide assay (MTT assay)

Human skeletal muscle myoblasts (HSMMs) were placed in a 6-well plate at a concentration of 6,250 cells/cm^2^ and were incubated in the SkGM™-2 Skeletal Muscle Cell Growth Medium-2 BulletKit™ supplemented with 10% fetal bovine serum (Lonza, Cat. CC-3245) at 37 °C in a 95% humidified atmosphere of 5% CO_2_. Cell viability of HSMMs was determined by the MTT assay (Sigma, Cat. TOX1-1 KT). In brief, HSMMs were plated at a density of 30,000 cells/cm^2^, respectively, in 96-well plates and incubated for 24 h. FSME (0–5,000 ng/ml) and ASME (0–5,000 ng/ml) were added for 24–72 h. After incubation, cells were treated with 3-(4,5-dimethyl-2-thiazolyl)-2,5-diphenyl-2H-tetrazolium bromide (MTT, 100 μL, 0.5 mg/ml) for 4 h at 37°C. The produced dark blue formazan crystals were solubilized by 100 μL DMSO. The absorbance at 570 nm was measured with a microplate reader. Cells without FSME or ASME treatment served as controls.

### 2.7 Lactate dehydrogenase (LDH) assay

Cell cytotoxicity of HSMM was performed by the LDH assay (Thermo Scientific, Cat. 88953). After the exposure of FSME and ASME treatment mentioned in the MTT assay, 10 μL of lysis buffer was added for 45 min at 37 °C. 50 μL of extracellular medium was transferred into a new 96-well plate and mixed with 50 μL of reaction mixture for 30 min at room temperature and protected from light. The absorbance at 490 and 680 nm was measured with a microplate reader. LDH activity was calculated by subtracting absorbance at 680 nm from the absorbance at 490 nm.

### 2.8 Animal DXA scan

To assess the changes in lean mass and fat mass upon FSME or vehicle treatment, dual energy X-ray absorptiometry (DXA, Ultrafocus 100, Faxitron) was performed for 20-month-old mice under general anesthesia by Ketamine-Xylazine (Ketamine: Saline: Xylazine = 3:2:3, 0.3 ml/kg, i. p.) on the day of termination. Analysis of body composition, including percentage of fat mass, soft weight, lean weight, and fat weight, was performed by the built-in BiopticsVision software after scanning.

### 2.9 Whole-body grip strength test

A grip strength meter (Ugo Basile, Cat. 47200) was used to measure whole-body grip strength. The assessment method of grip strength was according to the previous established protocol by Wang et al. (2020a). The mouse was made to grasp the grasping-grids; the peak tension in gram-force (gf) was recorded on a digital force transducer. The gauge was reset to 0 gf after stabilization. Peak tension was recorded by the gauge at the time the mouse released its whole-body limbs from the grids. Each mouse was measured three times, and the average of the three measurements was calculated. To minimize measurement error, three measurements were performed by the same assessor every 2 days. The grip strength data were normalized against the bodyweight of mice on the day of measurement.

#### 2.9.1 Histological assessment

The muscle embedding method was according to a previous established protocol by So (So, 1985). In brief, the gastrocnemius muscle was dissected and then coated with talcum powder evenly under optimum length for 20 s. OCT was added to the groove of the aluminum foil. The aluminum foil is plunged into liquid nitrogen immediately after placing the muscle tissue into the OCT. Frozen sections were taken out of the -80°C refrigerator to room temperature. The slides were rinsed in hematoxylin solution for 10 min. The rack holding the slides was placed in running water for 1 min to remove excessive hematoxylin, dipped in acid alcohol for 1 s, and then transferred back into running water for another 1 mins to remove acid alcohol. The slides were then put in Scott’s tap water for 3 min for bluing of hematoxylin and rinsed with distilled water to remove Scott’s tap water. After that, the slides were put into a 1% eosin Y solution for 5 min. After staining, the slides were dehydrated sequentially with distilled water (5 s), 70% ethanol (5 s), 80% ethanol (5 s), 90% ethanol (10 s), 100% ethanol (3 min), 100% ethanol (5 min), and clear ethanol in xylene for 8 min. To mount the slides, a drop of DPX mountant was placed over the tissue section and a coverslip was put on the top of the section to fully seal it.

#### 2.9.2 *Ex vivo* gastrocnemius muscle force and fatigability assessments

The assessment method was modified from Jiang et al. (Wang et al., 2020b). Under general anesthesia, the gastrocnemius muscle of the hindlimb was isolated and then mounted on a holder vertically to the dual-mode muscle lever arm system (300C-LR, Aurora Scientific Inc., Newmarket, Canada). The intact muscle was incubated in Ringer solution (121 mmol/L NaCl, 5.4 mmol/L KCl, 1.2 mmol/L MgSO_4_.7H_2_O, 25 mmol/L NaHCO_3_, 5 mmol/L HEPEs, 11.5 mmol/L Glucose, 2.5 mmol/L CaCl_2_) maintained at room temperature and continuously pumped with the gaseous mixture containing 95% O_2_ and 5% CO_2_. After the 5 min stabilization period, the muscle’s optimal length was measured after two tetanic contractions (1 A, 300 ms duration, 150 Hz stimulation frequency, and pulse width of 0.2 ms) with 5 min intervals. The optimal length of the gastrocnemius was determined prior to the subsequent test. The force frequency relationship was tested at different frequencies (25 Hz, 50 Hz, 100 Hz, 125Hz, and 150 Hz). The fatigue test was performed under optimal length. The fatigue analysis was tested by continuous stimulation (1 A, 300 ms duration, 150 Hz stimulation frequency, and pulse width of 0.2 ms) with 10 s intervals. Data analysis on force-frequency relationships and fatigue was done by 611A Dynamic Muscle Analysis (Mera et al., 2016) software.

### 2.10 Measurement of circulating cytokines

100 μL blood serum was taken from the cardiac puncture at the day of termination. Serum levels of IL-1a, IL-1b, IL-6, IL-9, IL-12 (p40), IL-17, IFN-g, MIP-1a, RANTES, TNF-alpha, G-CSF, KC, and Eotaxin were quantified with commercially available kits, Bio-Plex Pro Mouse Cytokine 23-plex Assay (Bio-Rad Laboratories, Incorporation, CA, United States). The panels were read with the Bio-Plex 200 system (Bio-Rad). The concentrations of the selected analytes were calculated according to the internal reference standard curve for each analyte.

### 2.11 Statistical analysis

All statistical analysis was performed with GraphPad Prism software 8.0 software (GraphPad, La Jolla, CA, United States). All data were presented as mean ± SD. The concentration of FSME and ASME underwent natural log transformation before the non-parametric Wilcoxon matched-pairs signed rank test in the cellular study. Animal study data statistical analyses were performed by the non-parametric Mann–Whitney test in DXA scan, muscle cross-sectional area and serum cytokines level. Comparison of muscle functions between groups was performed by the Wilcoxon matched pairs signed rank test, and the comparison between body weight and grip strength was performed by the multiple *t*-test, corrected by the Holm-Sidak test. A value of *p* < 0.05 was considered statistically significant.

## 3 Results

### 3.1 Protein identification and quantification

**FIGURE 2:**
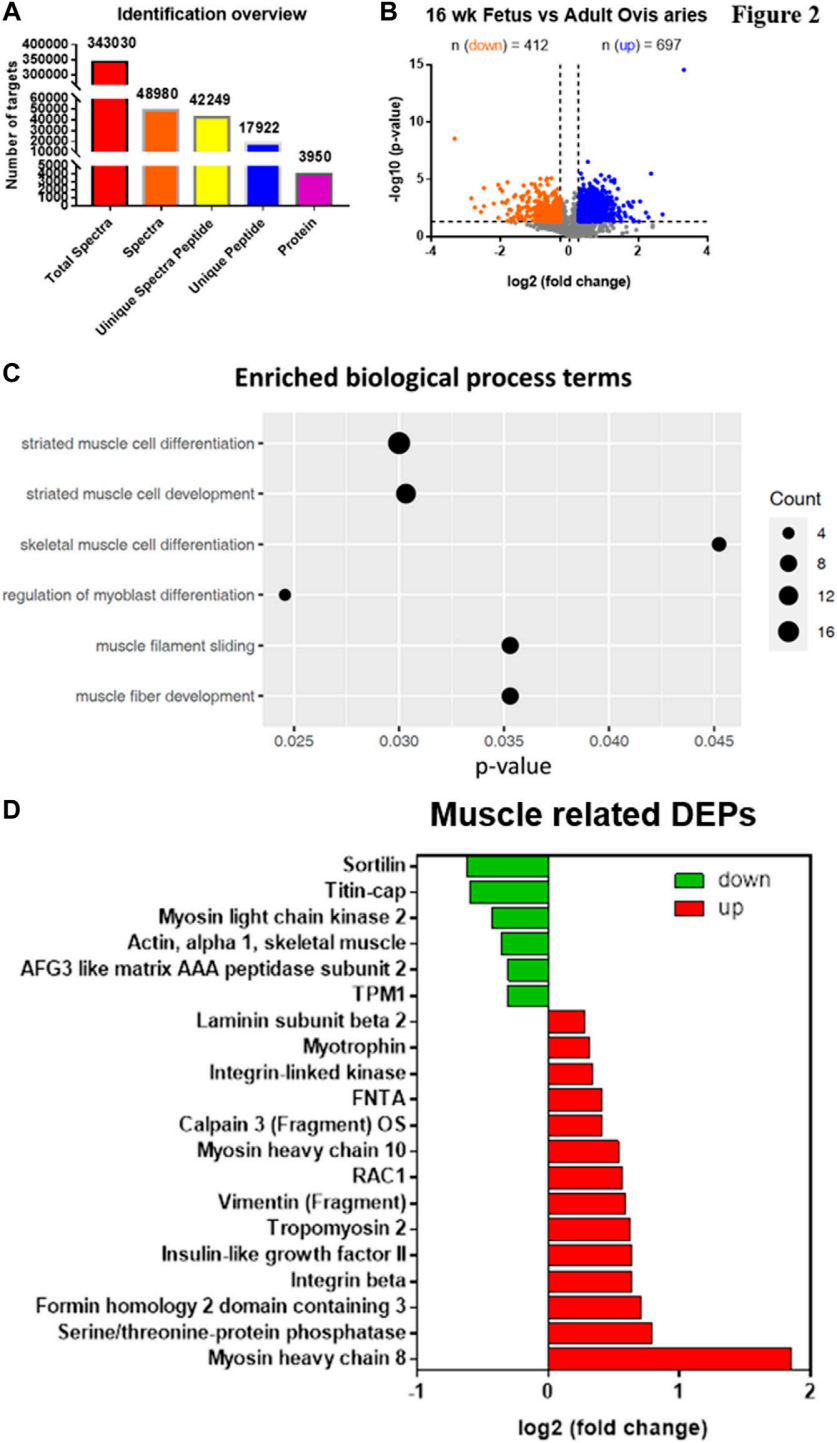
iTRAQ analysis of FSME and ASME proteomic expression profiles. **(A)** Analysis of the number of differentially expressed proteins between FSME 16-week-old fetal sheep and ASME of adult sheep. 3,950 proteins are identified in FSME and ASME. **(B)** Volcano plot of the relative quantitation results. A *t*-test was used between groups. Proteins with a ratio of <0.83 or >1.2 and *p* value < 0.05 were considered as differentially expressed proteins. Orange and blue plots indicate downregulated and upregulated differentially expressed proteins, respectively. Grey plots represent non-significant proteins. Amongst 3,950 proteins, 697 proteins are significantly upregulated while 412 proteins are significantly downregulated. **(C)** For the GO enrichment analysis, there are a total of 203 biological process terms enriched significantly. Among these, six of them are related to muscle or muscle lineage cells. Size represents the count of DEPs annotated to the GO terms. **(D)** In total, there are 20 DEPs annotated for the six GO terms. Green and red bars indicate downregulated and upregulated differentially expressed proteins, respectively.

An 8-plex LC-MS/MS analysis produced 343,030 spectra, which corresponded to 17,922 unique peptides, and 3,950 proteins were identified at a false discovery rate (FDR) of ≤0.01 (Figure 2A). We identified 3,950 proteins, of which 697 proteins are significantly upregulated while 412 proteins are significantly downregulated (Figure 2B).

### 3.2 Functional annotations of the up-regulated differentially expressed proteins (DEPs)

The 203 terms of Gene Ontology (GO) annotation for biological processes were enriched, and among these, six of them are related to muscle or muscle lineage cell, including skeletal muscle cell differentiation, regulation of myoblast differentiation, muscle filament sliding, and muscle fiber development. (Figure 2C). Fourteen myogenic proteins were present among the 20 up-regulated DEPs (Figure 2D).

### 3.3 FSME treatment promotes HSMM cell proliferation

**FIGURE 3:**
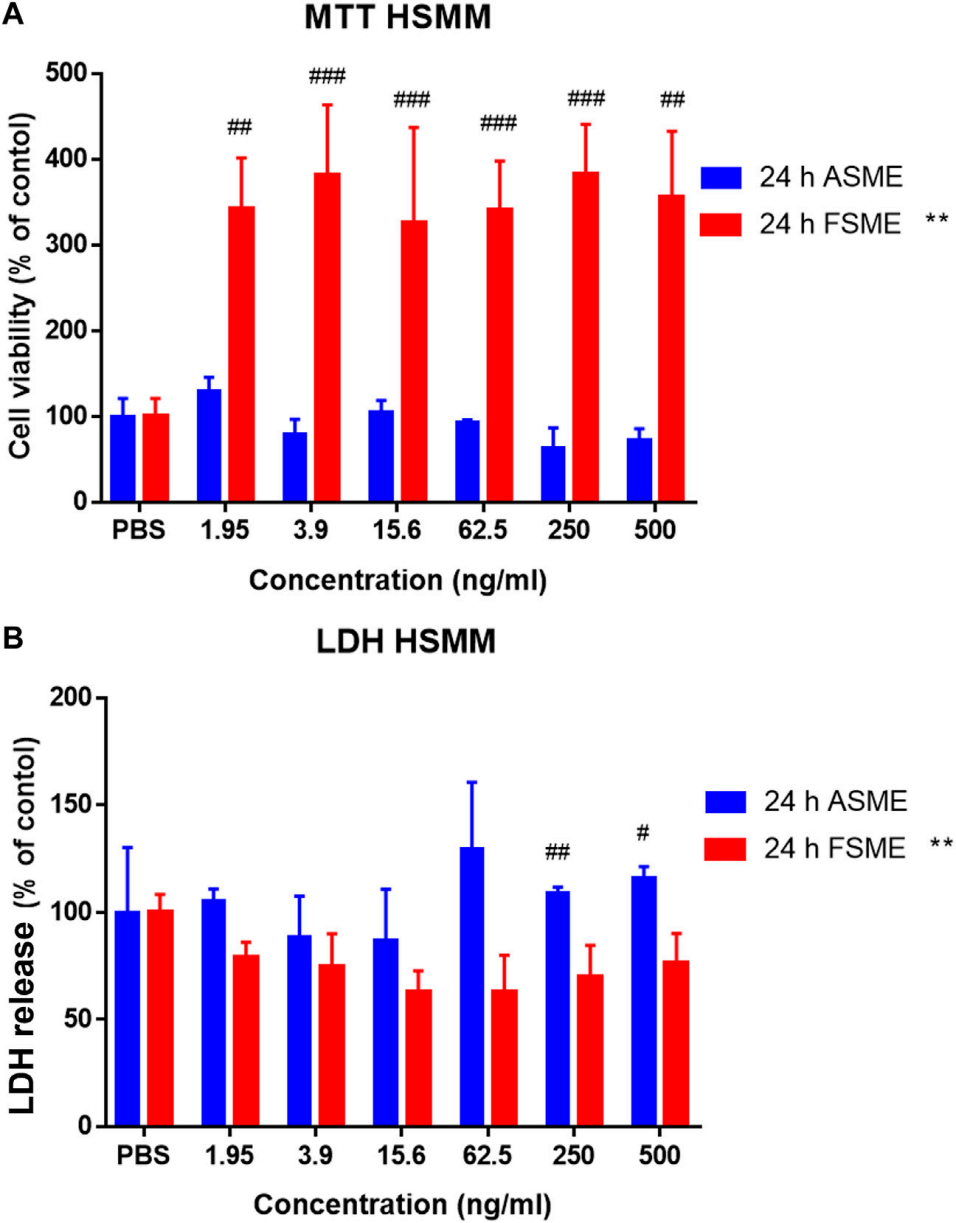
Cytotoxic effects of ASME and FSME in HSMM. **(A)** Cell viability was assessed by the MTT assay. Cells were incubated with increasing concentrations (1.95–500 ng/ml with a 2-fold increase) of ASME or FSME in a culture medium for 24 h. PBS was used as vehicle control. Results are expressed as the percentage of absorbance (the ratio of absorbance in ASME or FSME to that of PBS). **(B)** Effects of ASME and FSME on inducing plasma membrane damage in HSMM by LDH assay. HSMM cells were treated with PBS as vehicle control and ASME and FSME at 1.95–500 ng/ml (2-fold increase) for 24 h. After 24 h of ASME treatment, 250 ng/ml and 500 ng/ml significantly induced plasma membrane damage in HSMM. Data are means ± SD of three independent experiments. ** represents *p* < 0.01 indicates significance compared with FSME. ## represents *p* < 0.01 and # represents *p* < 0.05 indicate significance compared with control in ASME by one-way ANOVA, Dunn’s multiple comparisons test. N = 3; results are presented as mean ± SD.

As shown in Figure 3A, the FSME promoted HSMM cell proliferation in a concentration-dependent manner, while the ASME did not have any significant effect. The EC_50_ of FSME was 1.591 ng/ml in 24 h of treatment, while the EC_50_ of ASME could not be determined from the sigmoidal curve. Figure 3B shows the cytotoxic effects of the FSME and ASME on HSMM as determined by the LDH leakage assay. ASME induced cellular toxicity in HSMM at concentrations of 250 ng/ml and 500 ng/ml. On the contrary, FSME treatment did not induce significant cytotoxicity at all tested concentrations (0–500 ng/ml).

### 3.4 FSME treatment leads to higher body lean mass

**FIGURE 4:**
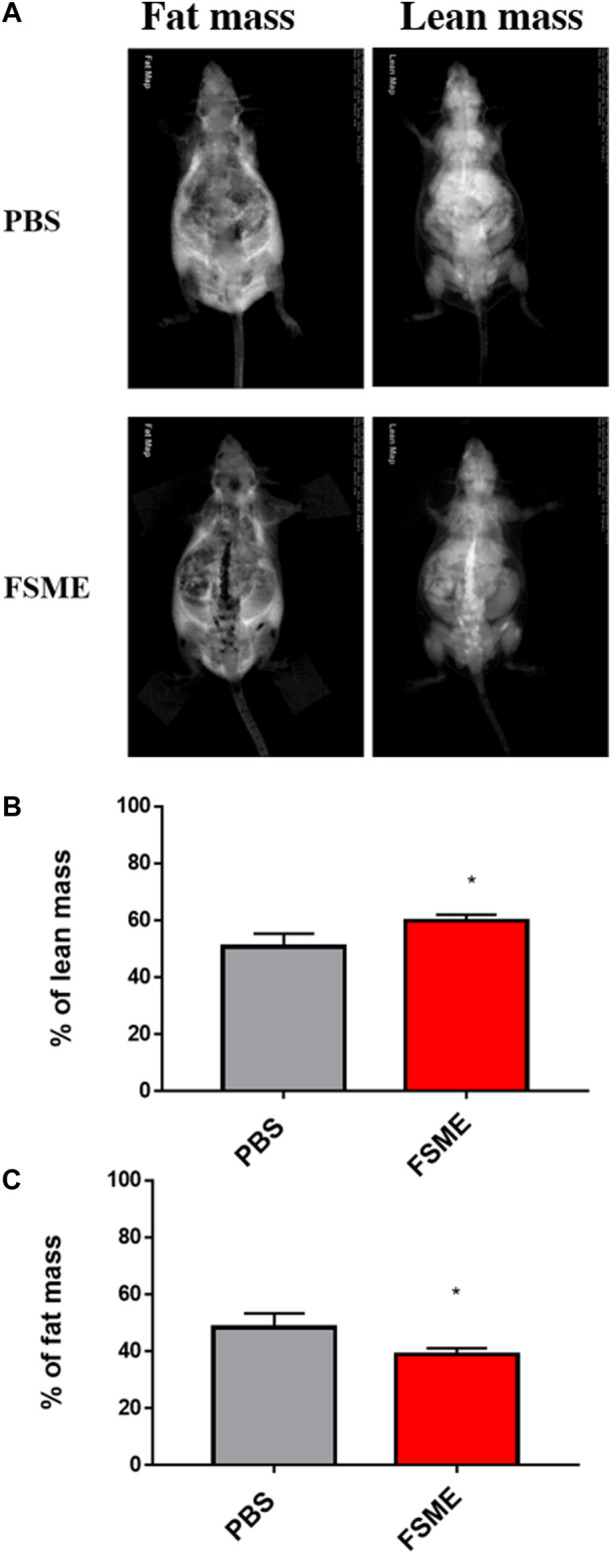
FSME group reduced fat mass and increased body lean mass in 20-month-old mice. **(A)** Representative DXA scan images of PBS or FSME mice. The left images are fat mass and the right images are lean mass. **(B,C)** Reduction in percentage of fat mass and increased in percentage of lean mass. * represents *p* < 0.05 by the Mann–Whitney test. N = 5; results are presented as mean ± SD.

The percentage of lean mass of the FSME group was significantly higher than the PBS group, at around 20% (Figure 4). On the other hand, the percentage of fat mass was significantly decreased in the FSME group by about 25%.

### 3.5 FSME treatment improves grip strength *in vivo*

**FIGURE 5:**
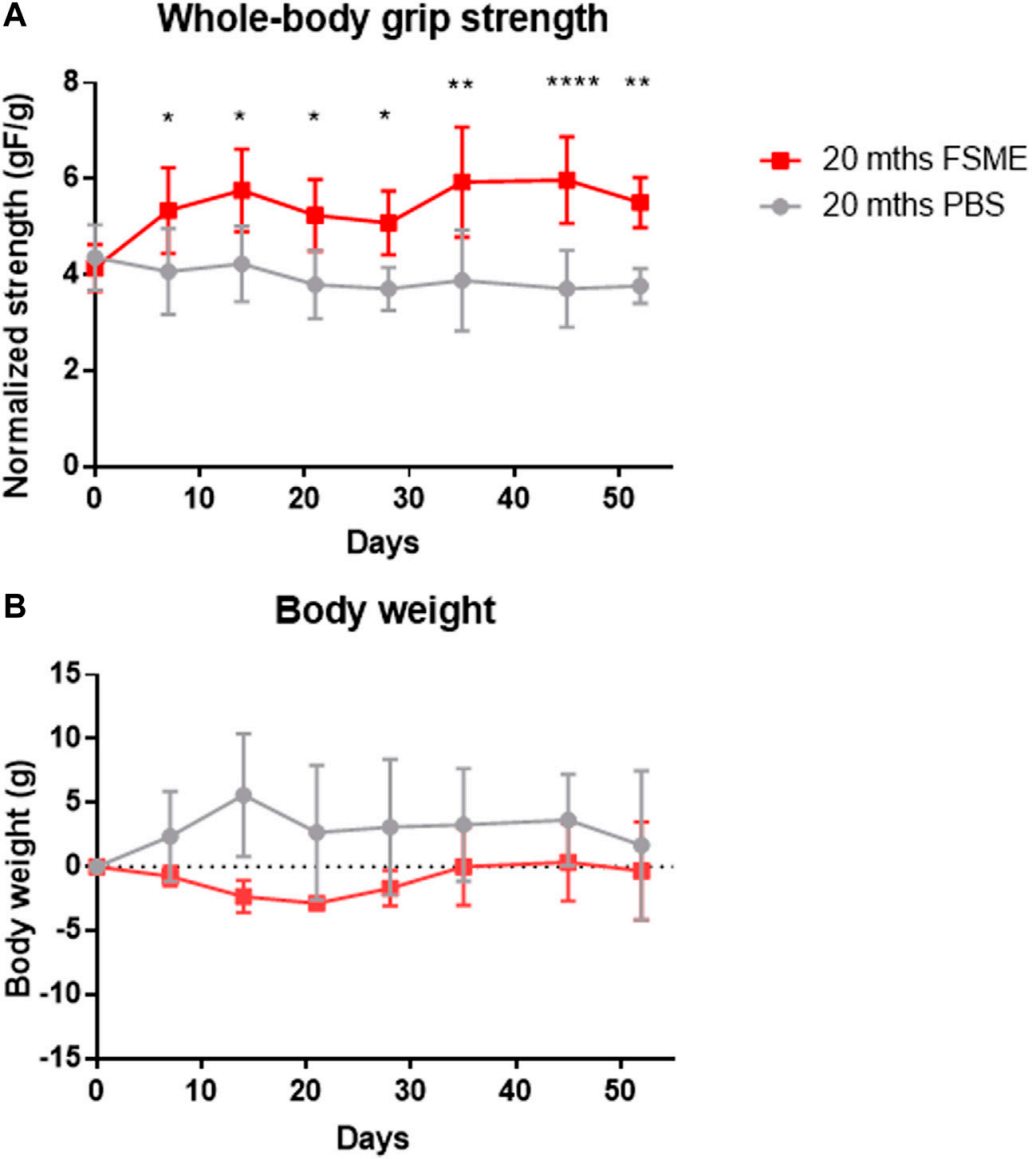
**(A)** Grip strength analysis of 20-month-old mice after FSME treatment for 8 weeks. FSME (30 mg/kg) administration is able to improve grip strength in FSME mice. **(B)** Body weight has shown no difference between the FSME group and PBS groups. Data are presented as mean ± SD, * represents *p* < 0.05, ** represents *p* < 0.01, and **** represents *p* < 0.0001 by the multiple *t*-test and Holm Sidak *post hoc* test.

*In vivo* assessment of the whole-body grip strength showed that the FSME group had higher grip strength relative to body weight throughout the study period (Figure 5A). At termination day, the grip strength in the FSME group was 50% higher than that in the PBS group (*p* < 0.01). The body weight of mice showed no significant difference between the two groups (Figure 5B).

### 3.6 FSME treatment reduces fatigability on gastrocnemius muscle *ex vivo*

**FIGURE 6:**
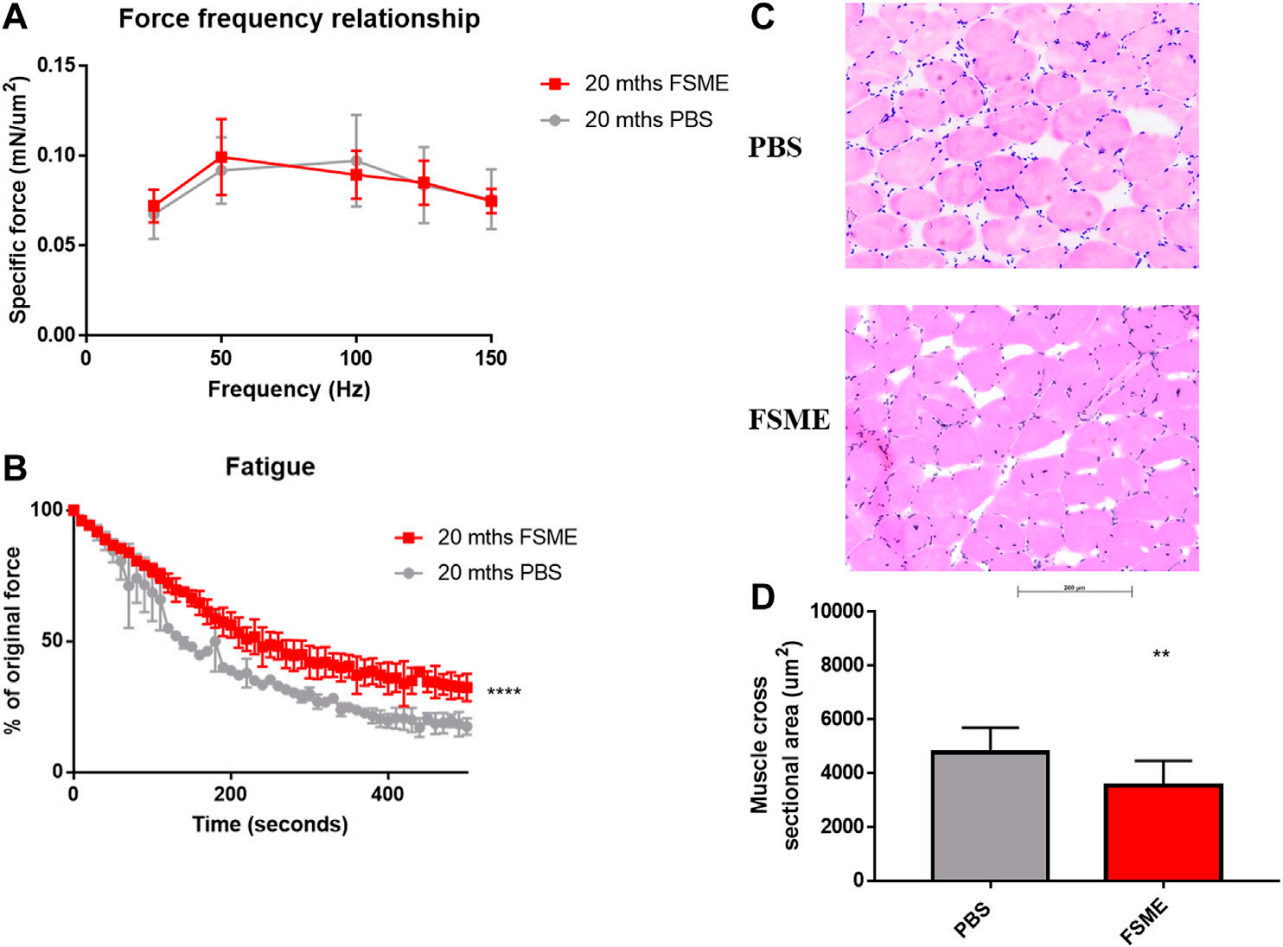
**(A)** Decrease in isometric twitch force of FSME treated mice under different stimulating frequency normalized with muscle cross section area. **(B)** Increased fatigue resistance of FSME treated mice gastrocnemius muscle. Results are presented as mean ± SD. Figure. **(C)** Representative IF staining and H&E staining of PBS and FSME group respectively. Figure. **(D)** Muscle cross-sectional area was larger in the PBS group. Data are presented as mean ± SD, **represents *p* < 0.01 by the non-parametric Mann–Whitney test method. N = 4; results are presented as mean ± SD.

To determine whether FSME treatment could improve muscle functional performance, the isometric force of the gastrocnemius muscle was measured using an *ex vivo* approach under different stimulating frequencies and fatigue analysis. In mice treated with FSME, there was no isometric force output difference between the two groups when normalized with the muscle cross section area (Figure 6A). However, when the gastrocnemius was subjected to a fatigue protocol, muscle from the FSME group showed greater fatigue resistance than that of the PBS group (Figure 6B). However, the muscle cross-sectional area is smaller in the FSME group (Figure 6D).

### 3.7 FSME treatment down-regulated inflammatory cytokines in the blood serum

**FIGURE 7:**
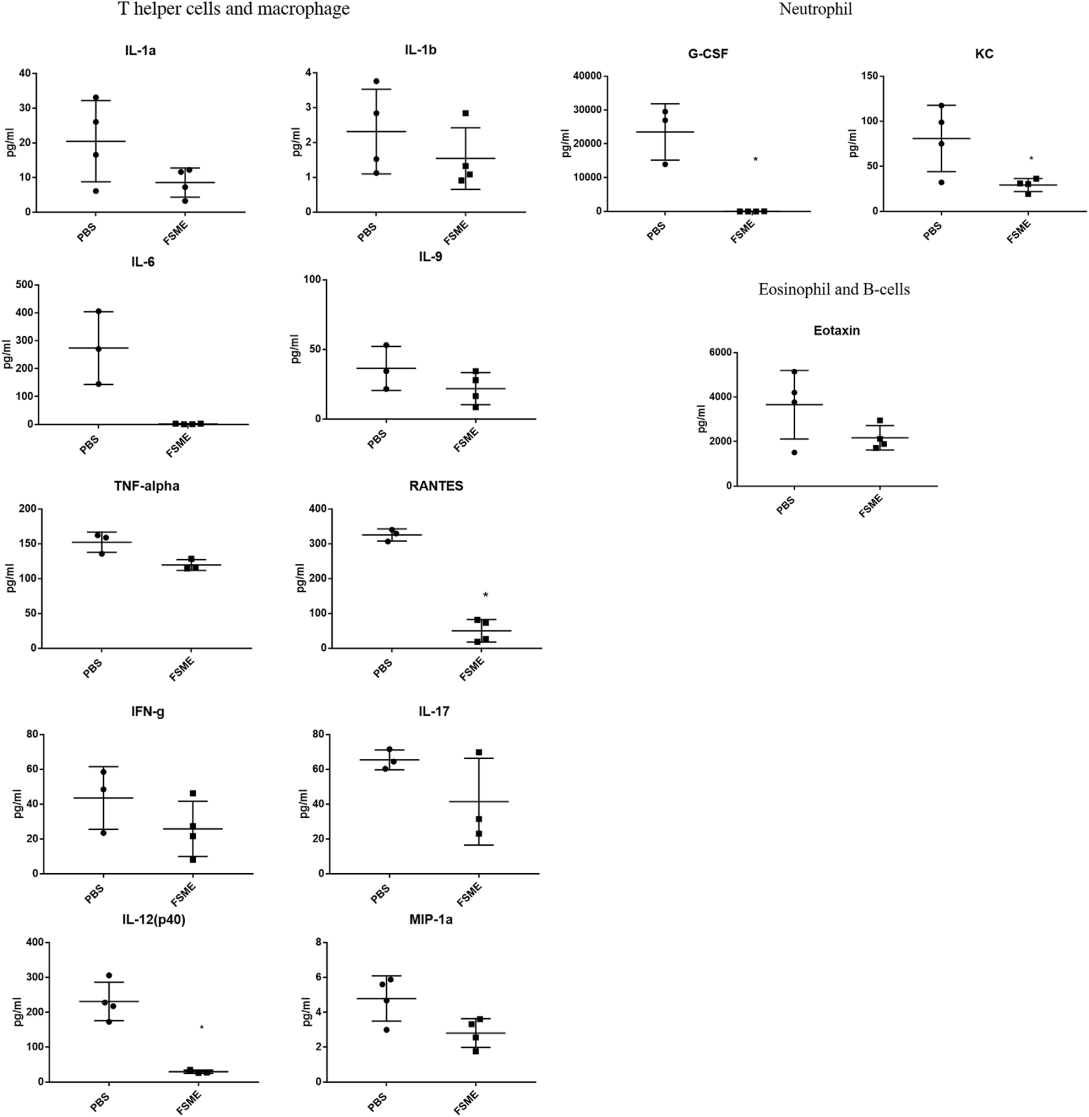
Multiplex analysis of serum inflammatory cytokines in 20-month-old mice after FSME (30 mg/kg) treatment for 8 weeks. Serum inflammatory cytokines showed no significant up-regulation in inflammatory cytokines after FSME administration in 20-month-old mice and pro-inflammatory cytokines, IL-12 (p40), RANTES, KC, and G-CSF show significant down-regulation after FSME treatment in 20-month-old mice. * represents *p* < 0.05 by the non-parametric Mann–Whitney test method. N = 4; results are presented as mean ± SD.

No significant up-regulation in inflammatory cytokines was observed in the 20-month-old FSME group compared with the PBS group (Figure 7). Interestingly, after FSME treatment for 8 weeks, we observed a significant drop in the circulating level of IL-12 (p40) from 90.8 ± 48.3 pg/ml to 82.65 ± 4.4 pg/ml, G-CSF from 23476 ± 8341.9 pg/ml to 28.35 ± 24.2 pg/ml, KC from 97.09 ± 21.2 pg/ml to 29.2 ± 7.2 pg/ml, and RANTES from 325.4 ± 17.3 pg/ml to 49.96 ± 32.1 pg/ml. The decrease in inflammatory cytokines in the FSME group indicates the likelihood that FSME treatment might modulate the circulating inflammatory profile. Of note, a statistical difference was not found in other measured cytokine markers.

## 4 Discussion

Our findings show that FSME treatment could promote the proliferation of human myoblast cells *in vitro* and result in increased lean mass and improved muscle strength, function, and grip strength in aged mice. Furthermore, multiplex analysis of serum cytokine levels demonstrated that FSME treatment did not result in an elevated inflammatory response in aged mice. Our findings demonstrate that muscle extract from fetal tissue consists of specific factors beneficial to muscle mass and strength in aging-associated muscle atrophy.

In the present study, the proteomic profiles and biological processes with muscle functions have been identified using proteomic analyses between FSME and ASME, providing new insight about distinctions in *in vivo* during embryonic muscle development. iTRAQ analysis revealed 697 up-regulated proteins and 412 down-regulated proteins in FSME when compared with ASME, suggesting there is a complex growth-related proteomic profile during the development of fetal skeletal muscle tissues. The results showed multiple differentially expressed myosin, tropomyosin and intermediate filament proteins were up-regulated, including MYH8, MYH10, VIM and TPM2 in FSME, which indicates a distinct regulation network of myofiber development underlying potential intervention for muscle related disorders (Zhang et al., 2016; Wang et al., 2017). The functional annotation results show that these proteins are associated with GO terms relating to skeletal muscle cell differentiation, regulation of myoblast differentiation, muscle filament sliding, and muscle fiber development. Tropomyosin is widely known as an actin-associated protein that regulates the actin filament function, as such, playing an important role in cell morphology determination and cellular process regulation, including protein transport and cell migration. Besides, myosin proteins play critical roles in cellular functions and biological processes. Different myosins are involved in the differentiation of muscle fibers; MYH8 encodes ATP hydrolysis (ATPase enzyme activity), actin binding and potential for kinetic energy transduction. A recent iTRAQ study also observed up-regulation in muscle-related DEPs, which annotated protein binding, muscle contraction enrichment in GO and KEGG analysis. Those metabolic and oxidative phosphorylation pathways are significantly associated with the development of muscle (Wang et al., 2020c).

It is believed that chronic inflammation in the muscle micro-environment can induce fibrosis and reduce muscle mass, which are closely associated with sarcopenia progression (Londhe and Guttridge, 2015). Being an endocrine tissue, muscle secretes a vast amount of myokines, including pro- and anti-inflammatory cytokines (Schnyder and Handschin, 2015), in response to stimulation such as exercise to modulate the immune system and elicit beneficial effects on bone health and mitochondria homeostasis (Scheffer and Latini, 2020). During fetal development, several growth factors and cytokines such as IL-10, IL-15, VEGF, and brain-derived neurotrophic factor are upregulated which are able to induce an anti-inflammatory response (Guzeloglu-Kayisli et al., 2009; Arrode-Brusés and Brusés, 2012; Redondo-Castro et al., 2017). It is reasonable to speculate that those growth factors and cytokines may exert beneficial effects on muscle, including pro-muscle innervation and muscle regeneration when administered to aged mice. In this study, apart from improving muscle function, we observed a significant down-regulation of several systemic pro-inflammatory cytokines, including KC, RANTES, G-CSF, IL-6, and IL-12. RANTES and IL-6 are also classified as myokines, which are synthesized and released by myocytes during muscular contractions. Similar to our findings, a human study revealed higher expression levels of IL-6 and IL-12 in nonagenarians compared with young controls (Palmeri et al., 2012). IL-6 and IL-12 serum levels were found to correlate with the onset of frailty, poor physical performance, and loss of muscle strength (Rong et al., 2018; Chen et al., 2021). Participants with more severe sarcopenia had lower IL-6 and IL-12 (Mikó et al., 2018). In a clinical study, the relationship between IL-12 and age-related muscle loss may be an early diagnosis indicator for sarcopenia in the elderly (Chen et al., 2021). Natural aging was positively correlated with an increase in RANTES (Mansfield et al., 2012). In addition, the secretion of RANTES impaired skeletal muscle regeneration upon injury in mice (Kohno et al., 2011). The down-regulation of IL-6 and RANTES after administration of FSME may imply that FSME has a potential effect on preserving muscle strength or delaying the onset of frailty. As inflammatory cytokines have a negative impact on skeletal muscle growth and lead to muscle atrophy (Mera et al., 2016), the decrease of serum inflammatory cytokine levels indicated that FSME might prevent muscle from atrophy and fibrosis. FSME may not only elicit beneficial effects localized at the injection site but systemically affect the whole body. Our results have shown FSME improves fatigue-resistant in *ex vivo* gastrocnemius muscle function and restores whole-body muscle performance and function as reflected by the whole-body grip strength.

Given that muscle weakness comes with age progression, any means to enhance muscle fatigue resistance has a beneficial implication for overcoming mobility impairments in the elderly population (Callahan et al., 2009). In our intervention, injection of FSME could enhance fatigue resistance compared with the PBS group, as indicated by *ex vivo* fatigue analysis. Fatigue resistance has been considered as an essential parameter for muscle health in the elderly population (Katsiaras et al., 2005). Higher fatigue in muscles could impair physical function (in terms of gait speed) and increase the risk of falls in older men (relative risk = 1.25, 95% CI: 1.14–1.36) over 3 years of follow-up, a study in the United States (Renner et al., 2020). A clinical observation in geriatric falls showed that muscle fatigue has a deleterious effect on the coordination of reactive postural control and increases the risk of falls (Papa et al., 2015). Higher fatigue resistance protects the elderly from falling and establishes a healthier aging lifestyle (McLeod et al., 2016). Reduction in fatigue might reduce risk of fall and reduce geriatric hospitalizations in fall-related injuries. The effect of FSME on fatigue resistance sheds light on the development of novel pharmacological treatment options for reducing fall risk.

As indicated previously by numerous studies, primary sarcopenia is an age-related condition characterized by a progressive loss in muscle mass, strength, and performance. Diagnosis of sarcopenia in clinical setting include the use of DXA scan, gait test, hand grip test (Lera et al., 2020), Short physical performance battery (SPPB) (comprises of a combination of gait speed, a balance test, and a chair stand test) and 400-m walk or long-distance corridor walk (Beaudart et al., 2016; Cruz-Jentoft et al., 2019). Sarcopenic patients were generally found to perform less well in the sarcopenia/frailty tests (Beaudart et al., 2016; Cruz-Jentoft et al., 2019). It was clearly shown in our study that FSME elicits improvement on the grip strength test in aged mice, thereby highly indicating that FSME administration could potentially improve muscle performance. In addition to the grip strength test, a reduction in the percentage of fat content examined by DXA scan was also observed upon FSME treatment. A previous study showed that fat infiltration is associated with both metabolic and mobility impairments in geriatric populations (Marcus et al., 2010). A further to the mobility impairment, an increase in fat content is also linked to inflammation (Erskine et al., 2017). Consistent with our finding on cytokines level, both pro-inflammatory cytokines and fat content reduce upon FSME treatment. Nonetheless, the finding of FSME’s ability to reduce fat content *in vivo* could be a potential attributor to muscle performance via the reduction in age-associated inflammation. However, the detailed underlying mechanism remains to be elucidated.

In summary, we identify the presence of several DEPs during muscle development in the sheep fetus and report that local administration of FSME enhances systemic muscle quality function in aged mice. A detailed mechanism study is warranted to characterize the putative effect of FSME on improving the muscle developmental quality. The current observations serve as a foundation for developing novel potential therapeutic agents for sarcopenia.

## Data Availability

The original contributions presented in the study are included in the article/Supplementary Material; further inquiries can be directed to the corresponding author.
